# First report card on physical activity for children and adolescents in Slovakia: a comprehensive analysis, international comparison, and identification of surveillance gaps

**DOI:** 10.1186/s13690-024-01241-4

**Published:** 2024-01-30

**Authors:** Peter Bakalár, Lenka Hnidková, Beáta Ružbarská, Pavel Ružbarský, Terézia Kovalik Slančová, Jaroslava Kopčáková, Michaela Kostičová, Aleš Gába

**Affiliations:** 1 University of Presov, Faculty of Sports, Prešov, Slovakia; 2 Pavol Jozef Šafárik University, Faculty of Medicine, Košice, Slovakia; 3 Palacký University Olomouc, Olomouc University Social Health Institute, Olomouc, Czech Republic; 4 Comenius University, Faculty of Medicine, Bratislava, Slovakia; 5 Palacký University Olomouc, Faculty of Physical Culture, Olomouc, Czech Republic

**Keywords:** Active play, Organised sports, Active transportation, Sedentary behaviours, Physical fitness, Sleep

## Abstract

**Background:**

National surveillance of physical activity (PA) is essential to establish public health policy actions for PA promotion and evaluation, thereby promoting accountability. The main goal of this study is to comprehensively analyse surveillance data on PA behaviours, outcomes, and influencing factors among Slovakian children and adolescents by using the methodology of the Active Healthy Kids Global Alliance Global Matrix (AHKGA-GM) project. Secondary goals are to provide comparisons with international data and the identification of surveillance gaps.

**Methods:**

A comprehensive multilevel search strategy for data about 10 core indicators and 1 additional indicator published between 2015 and 2020 (solely pre-COVID-19-pandemic data) was used. The data were then synthesised, and a set of standardised benchmarks was used to assign grades according to The Global Matrix 4.0 Grading Rubric.

**Results:**

A total of 552 potentially relevant data resources were retrieved, of which 34 were identified as eligible for data extraction. Grade B was assigned to four core indicators, grade C to five core indicators, and grade D to one core indicator. The additional *Sleep* indicator was graded a C–. Compared with the average grades from countries with high Human Development Index scores, Slovakia received higher grades in five core indicators (*Overall Physical Activity*, *Active Transportation*, *Sedentary Behaviours*, *School*, *Government*), and in the aggregate *Behavioural* average and *Overall* average. Compared with global averages, Slovakia had higher grades in the aforementioned core and aggregate indicators, and in the *Community and Environment* core indicator and aggregate *Sources of Influence* average indicator. Numerous surveillance gaps were identified.

**Conclusions:**

The overall grading of the available surveillance data suggests the need for improvement in all 10 surveilled core indicators, and in additional *Sleep* indicator. Despite the fact that numerous identified surveillance gaps limit the overall informative value of the current grade, they provide the important information needed to enhance surveillance of PA-related indicators in Slovakia. For instance, the focus should be put on younger children and parents, on obtaining the device-measured data on various movement behaviours, on the topic of outdoor physical activities, and policy evaluation.

**Supplementary Information:**

The online version contains supplementary material available at 10.1186/s13690-024-01241-4.

**Table:** 

**Text box 1. Contributions to the literature**
• Our research is first of its kind in Slovakia and brings important information needed to enhance the surveillance of physical activity behaviours, outcomes, and influencing factors (indicators) in Slovakian children and adolescents.
• Even though the grade for *Overall Physical Activity* indicator was higher compared to average grade of this indicator in a group of countries with high Human Development Index, the analysis suggests the need for improvement in surveillance itself and in policy making.
• Surveillance gaps such as lack of the data for younger children and parents and for device-measured data on movement behaviours limit the informative value of currently available surveillance data.

## Background

A large body of evidence has already established that increased amounts and higher intensity levels of physical activity (PA) in children and adolescents are associated with many beneficial health outcomes [1]. For instance, PA was found to be positively associated with bone health [2–4], cardiorespiratory fitness [5, 6], brain activation and microstructural plasticity [7], self-rated health [8], well-being and quality of life [9], and negatively associated to adiposity and cardiometabolic risk score [5].

The most recent pooled analysis reveals that in 2016 the global prevalence of insufficient physical activity in adolescents was 81% (77,6% of boys and 84,7% of girls) [10].

To change this unfavourable situation, the World Health Organisation (WHO) proposed in its Global Action Plan on Physical Activity 2018–2030 (GAPPA) to implement various effective evidence-based policies [11], but as the WHO reported, progress in national implementation of recommended GAPPA policy actions has been slow globally. Only two GAPPA indicators showed implementation by over three quarters of all countries - conducting national surveillance of PA among adults, and among children and adolescents being one of them [12]. National surveillance of PA is essential to establish public health policy actions for PA promotion and evaluation, thereby for promoting accountability [13]. Although national and international standardized surveillance of PA among children and adolescents has increased in recent years, challenges for the global surveillance of PA persist. For example, there are substantial inconsistencies across/within included initiatives, resulting in varying estimates of the PA estimates of children and adolescents at the global, regional and national levels [14]. And, although 75% of countries worldwide have some type of PA surveillance system for children and adolescents, the data are often more than 5 years old, and 10% have data that are more than 10 years old [12]. This indicates the need for a step change in PA surveillance [15].

The AHKGA-GM project is one of the current efforts toward the development of a comprehensive system for the surveillance of PA behaviours, outcomes, and influencing factors (hereafter called indicators) in children and adolescents. In a form of so-called Report Card (RC), it summarises the best available evidence on various PA indicators taking in the account all its dimensions, and facilitating various types of data analyses [14]. By doing so it has impact on raising the awareness and capacity building in the national and international scientific community, disseminating information to the general population and stakeholders, and on powering the movement to get children and adolescents moving [16]. After successful AHKGA-GM RCs releases at events in Toronto, Canada (Global Matrix 1.0, 15 countries, 2014), Bangkok, Thailand (Global Matrix 2.0, 38 countries, 2016), and Adelaide, Australia (Global Matrix 3.0, 49 countries, 2018), the last release of Global Matrix 4.0 took place in Abu Dhabi, United Arab Emirates in 2022 with 57 countries and regions participating [17]. The later was the first time when Slovakia participated in AHKGA-GM project. This study summarises its outcomes in Slovakia and is meant to serve as a comprehensive evidence-based source of information for various stakeholders. As the Tremblay et al. [17] conclude, specific country RCs have been shown to be very effective and influential across multiple sectors in terms of transferring interventions, policies, and practices, improving surveillance, and advocacy purposes. For instance, solely between the years 2004 and 2014 Canadian RC has achieved > 1 billion media impressions, distributed > 120,000 printed copies and > 200,000 electronic copies, and benefited from a collective ad value > $10 million [18].

Therefore, the main goal of this study is to comprehensively analyse surveillance data on PA behaviours, outcomes, and influencing factors among Slovakian children and adolescents by using the methodology of the AHKGA-GM project. Secondary goals are to provide comparisons with international data and the identification of surveillance gaps.

## Methods

The process of developing the RC was in line with the harmonised methodology used by the 57 countries and regions that participated in the AHKGA-GM 4.0 project [19]. The national research work group (RWG) comprised eight members: PA experts from the University of Prešov (Slovakia) and Palacký University Olomouc (Czech Republic) and public health experts from Pavol Jozef Šafárik University (Slovakia) and Comenius University (Slovakia).

### Search strategy

A comprehensive multilevel search strategy originally developed by the Czech RC RWG [20] was used to search for published and unpublished data collected between 2015 and 2020 that focused on 10 core indicators (*Overall Physical Activity, Organised Sports and Physical Activity, Active Play, Active Transportation, Sedentary Behaviour, Physical Fitness, Family and Peers, School, Community and Environment*, and *Government*) and one additional indicator (*Sleep*) among Slovakian children and adolescents (6–17 years old) (Additional Table 1). The search was focused solely on the pre-COVID-19-pandemic data to avoid affecting the data by pandemic and to allow for comparisons of the pre-pandemic, pandemic and post-pandemic data in the next AHKGA-GM project editions. The first case of COVID-19 was officially reported in Slovakia on March 6th, 2020. The search strategy consisted of two database searches (Medline Ovid and Medvik), searches for grey literature and unpublished data (via Google and by contacting colleagues outside the RWG), and a hand search of key journals (Slovak journal “Telesná výchova a šport” and Czech journal “Tělesná výchova a sport mládeže”). The search process was terminated in May 2021.

### Screening

**Fig. 1:**
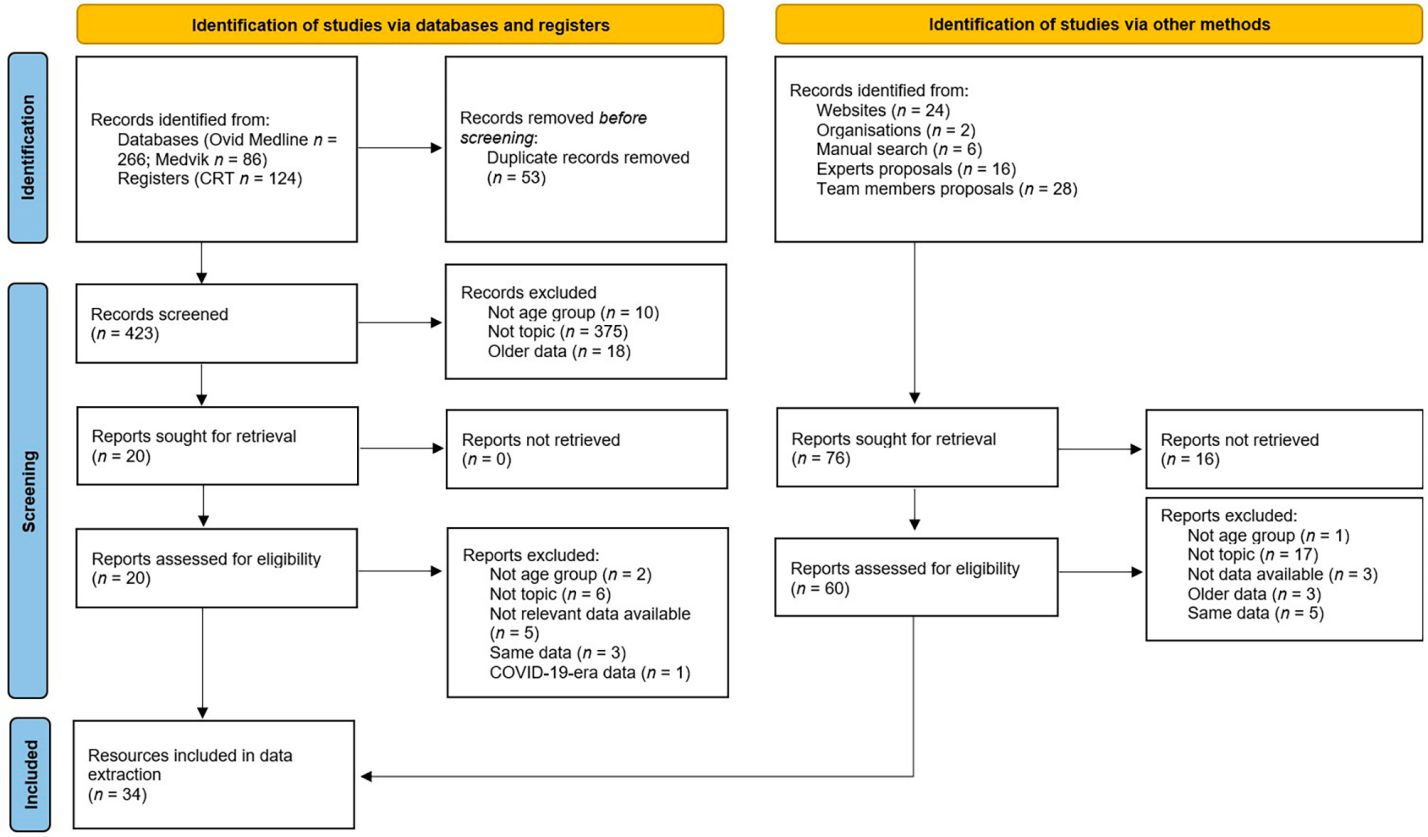
Flow diagram of search strategy for identifying all available resources. Legend: CRT – Central Register of Theses

The RWG conducted a comprehensive analysis of all retrieved and potentially relevant resources. The eligibility of the resources was evaluated by RWG members by providing two independent screenings (one by lead author and the other by various RWG members). In cases of disagreement between the two eligibility decisions, a third decision by another RWG member was considered. This process was completed in June 2021 (Fig. 1).

### Quality assessment

The methodological quality of each data resource was evaluated using a non-standardised nine-item assessment tool that consisted of nine questions related to the participant recruitment method, sample size, proportion of girls, age range, and validity of the method used, with response options of ‘yes’, ‘no’, and ‘unclear’. Except for government documents, the data sources were scored according to the degree to which the specific criteria were met (0 = ‘no’, 1 = ‘yes’). A quality score was calculated for each data source and used to classify the level of methodological quality as follows: high (7–9 points), medium (4–6 points), low (2 or 3 points), and insufficient (< 2 points) quality [21]. Only data resources of low to high quality were included in the data extraction.

### Data extraction and grading of indicators

Data extraction was performed to provide a body of relevant data that best aligned with the 10 core indicators and 1 additional indicator. Additional Table 1 presents the definitions and associated benchmarks for each indicator. If data were available for benchmarks of multiple indicators, the weighted mean (adjusted for sample size) of the benchmark was used. For the *Government* indicator, the evaluation process involved the use of the Health-Enhancing Physical Activity Policy Audit Tool (version 2) and grading rubric published by Ward et al. (2021) [22]. For the *Sleep* indicator, the evaluation process was based on the National Sleep Foundation sleep time duration recommendations for children (6 to 13 years old) and adolescents (14 to 17 years old) [23], which was one of the proposed benchmarks. Subsequently, each indicator was assigned a grade using the following harmonised grading rubric: A+ (94–100%), A (87–93%), A– (80–86%), B+ (74–79%), B (67–73%), B– (60–66%), C+ (54–59%), C (47–53%), C– (40–46%), D+ (34–39%), D (27–33%), D– (20–26%), and F (< 20%). The indicator was graded as ‘INC’ (incomplete data) in cases lacking relevant data as determined in the evaluation. This process ended in January 2022. The draft RC, along with the rationale for the grades, were audited by members of the AHKGA Executive Committee and approved in February 2022.

## Results

Using a multilevel search strategy, a total of 552 potentially relevant data resources were identified, of which 476 were records from databases and registers and 76 were identified by other methods. After removing 53 duplicates, the titles and abstracts of the remaining search results were screened. Based on content and quality, a final sample of 34 relevant resources was used for data extraction (Fig. 1). Eleven indicators were graded using 17 of the 31 proposed benchmarks (55%). More than half of the data resources (*n* = 19; 61%) were from the Health Behaviour in School-Aged Children Study (HBSC) (published or unpublished data). Nine resources (29%) were used to grade the *Government* indicator using the grading rubric published by Ward et al. (2021) [22]. A list of data resources is presented in Additional Table 2.

The corresponding grades for the 10 core indicators included in the RC are listed in Table 1. Grade B was assigned to four indicators, grade C to five indicators, and grade D to one indicator. The grades for the three aggregate indicators were calculated using the same method as Aubert et al. (2022) [19] as follows: (1) behavioural indicators, C– (average grade for the *Overall Physical Activity*, *Organised Sports and Physical Activity*, *Active Play*, *Active Transportation*, and *Sedentary Behaviour* indicators); (2) *Sources of Influence* indicators, C+ (average grade for *Family and Peers*, *School*, *Community and Environment*, and *Government* indicators); and (3) overall average indicator, C (average grade of the 10 core indicators). Furthermore, the *Sleep* indicator was graded a C–.

Compared with the average grades of a group of countries with high Human Development Index (HDI) scores, Slovakia had higher grades for five core indicators (*Overall Physical Activity*, *Active Transportation*, *Sedentary Behaviour*, *School*, *Government*) and for the aggregate *Behavioural* average and *Overall* average (Table 1). Compared with the global averages, Slovakia had higher grades in the same core and aggregate indicators as previously mentioned, and in the *Community and Environment* core indicator and aggregate *Sources of Influence* average indicator (Table 1). Notably, the varied surveillance methods used among countries included in the AHKGA-GM project make comparisons among countries challenging and often invalid [15]. Therefore, these results should be considered with caution.

**Table 1:** Slovakia’s Global Matrix 4.0 grades compared with average grades from a group of countries with high Human Development Index cores and global averages

Indicator	Slovakia’s grades	Average grades for countries with high HDI scores^a^ (*n* = 42)	Average Global Matrix grades^a^ (*n* = 57)
*Overall Physical Activity*	B–	D+	D
*Organised Sports and Physical Activity*	C–	C	C–
*Active Play*	C–	C–	C–
*Active Transportation*	C	C–	C–
*Sedentary Behaviour*	C–	D	D+
*Physical Fitness*	D+	C	C–
*Family and Peers*	C–	C–	C–
*School*	B	B–	C+
*Community and Environment*	B–	B–	C+
*Government*	B–	C+	C
*Behavioural average*	C–	D+	D+
*Sources of Influence average*	C+	C+	C
*Overall average*	C	C–	C–

^a^Adapted from Aubert et al. 2022 [19]

## Discussion

To comprehensively analyse the available surveillance data on PA behaviours, outcomes, and influencing factors in Slovakian children and adolescents, we used the AHKGA-GM project methodology. Data from 34 relevant resources were extracted for grading the 10 core indicators and 1 additional indicator. The overall average of the 10 core indicators was graded a C, which suggests room for improvement in all 10 core indicators.

### Overall physical activity (B–)

The grade for the *Overall Physical Activity* indicator was based on the questionnaire data from the WHO’s collaborative HBSC, which was conducted in 2018 in Slovakia (*n* = 8710; [24]). Approximately two-thirds (66%) of adolescents met the PA recommendation, with higher compliance with the recommendations observed in younger adolescents (< 14 years old; 68%) compared with older adolescents (> 14 years old; 62%). This corresponds to the grading B– according to harmonised grading rubric. Boys (70%) reported meeting this recommendation more often than girls (62%). A similar result was observed in younger versus older adolescent boys and girls (71% and 68% in boys vs. 65% and 56% in girls). The high percentage of adolescents who complied with the recommendations is the result of the cut-off value used, which was based on the comparative analyses of questionnaire and device-measured PA data [25]. The results suggest that the previously used cut-off value for self-reported PA data underestimated compliance with recommendations by nearly three times (66% vs. 23%). The lack of device-measured PA data is therefore the main limitation identified in the analysis of data for this indicator. Furthermore, an absolute gap in the data was identified for children aged 6–9 years. Therefore, to improve the surveillance of *Overall Physical Activity*, we recommend the use of device-based measurements of PA in both children and adolescents to obtain more accurate data on PA levels.

### Organised sports and physical activity (C–)

Similar to the previous indicator, data from the HBSC were used to grade the *Organised Sports* and *Physical Activity* indicators (unpublished data). Data for organised team sports (*n* = 8574) and organised individual sports (*n* = 8392) were used to calculate the average grade. Overall, 41% of adolescents participated in organised sports, with 51% participating in organised team sports and 31% in organised individual sports. Boys reported participating in organised team sports more often than girls (62% vs. 40%), as did younger (64% vs. 41%) and older adolescents (56% vs. 38%). In contrast, girls reported participating in organised individual sports more often than boys (35% vs. 27%), mainly owing to the higher prevalence of participation in organised individual sports reported by younger girls compared with boys (41% vs. 27%). The prevalence of participation in organised individual sports was similar in older adolescent girls and boys (25% vs. 26%). As these data suggest, half of the Slovakian adolescent population does not participate in organised sports. This should be addressed within PA promotion activities, since organised sports may play a significant role in meeting PA recommendations in children and adolescents [26]. Meanwhile, since a gap in the data for children was identified for this indicator, we recommend focusing on this age group in future surveillance efforts.

### Active play (C–)

The *Active Play* indicator was graded using data from only one pilot study (*n* = 625; [27]) and only one of the two proposed benchmarks. Therefore, the grade for this indicator should be considered with caution. In the aforementioned study, nearly 44% of children under 9 years of age reported being outdoors for more than 2 h per day, and boys reported being outdoors more often than girls (47% vs. 41%). The reported proportions were rather low considering the growing evidence of physical, mental, and social benefits of outdoor activities [28–32]. The main limitation of grading this benchmark was the use of questionnaire-type data. Among the identified gaps was the lack of data for the second benchmark and other age groups. Improved surveillance within this indicator may therefore require device-based measurement of different types of PAs (unstructured/unorganised and outdoor) in various age groups.

### Active transportation (C)

Unpublished data from the HBSC (*n* = 1062) indicated that 49% of adolescents used active modes of transportation to school, with a similar prevalence observed in girls (50%) and boys (48%). Younger adolescent girls reported using active modes of transportation more often than boys (54% vs. 49%), although in older adolescents the opposite was observed, with boys reporting more frequent use of active modes of transportation (48% vs. 43%). When analysing published data from the HBSC [33] one particular pattern was observed within this indicator, namely that more adolescents walked home from school versus to school. This could be the result of parents driving adolescents to school in the morning. A data gap was identified for children; therefore, we recommend collecting surveillance data for this age group as well.

### Sedentary behaviour (C–)

The grade for the *Sedentary Behaviour* indicator was based on unpublished HBSC composite data from the HBSC, which consisted of watching television, digital video discs, or videos (including videos on the Internet, e.g. YouTube) (*n* = 1053) and playing games on a personal computer (PC), play station, or phone for less than 2 h per weekday (*n* = 1052). Among 10- to 16-year-olds, 44% met the screen time recommendation. Girls (62%) reported compliance with the screen-time recommendation twice as often as boys (31%), with similar results observed in younger (61% vs. 32%) and older (62% vs. 30%) adolescents. The grading of this indicator was limited in that the data used did not fully reflect the wording of the recommendations. Other possible resources of data were not used for the analyses for the same reason since they solely focused either on time spent on the Internet or PC without differentiating between potentially beneficially and not beneficially (e.g. studying) time spent in front of the screen. Therefore, we recommend unifying the surveillance method for this indicator to reflect the recommendations. Moreover, another limitation existed owing to the use of questionnaire-based data. Therefore, we recommend obtaining device-based measurements of sedentary behaviour, which could promote understanding of the movement behaviours of children and adolescents. Similar to previous indicators, a gap in the data was identified for children; therefore, we recommend focusing on this age group during surveillance.

### Physical fitness (D+)

In accordance with the benchmark, the *Physical Fitness* indicator was graded based on the average number of laps completed within the 20-metre shuttle run. Data on 9- to 15-year-old children and adolescents from four resources (*n* = 4209; [34–37]) were weighted and assessed against age- and sex-specific international normative data. This led to placement of the Slovak children and adolescents on 38th percentile (35th in boys and 40th in girls). The grading of this indicator was limited in that it was not possible to include the results of the first mandatory nationwide assessment of the physical abilities of first graders in 2018 (*n* = 38 690; [38]) because the international normative data were only available for 9-year-olds. Another limitation was caused by the coronavirus disease pandemic, which restricted the nationwide assessment of the physical abilities of third graders planned for the year 2020. However, since the assessment of first and third graders will be performed annually, it will provide data for the surveillance of the *Physical Fitness* indicator in the future. Moreover, since a gap in the data was identified for other age groups, we recommend expanding the assessment to all elementary school grades. This recommendation is currently being considered within the legislative process of the Slovak National Council, based on the initiative of RWG members.

### Family and peers (C–)

One benchmark was used to grade the *Family and Peers* indicator, which reflects tangible parental support (i.e. doing activities with their children). Based on unpublished data from the HBSC, 39% of adolescents reported that their parents played sports with them (*whole sample* = 6179), and 54% of adolescents reported that their parents took walks with them was 54% (*whole sample* = 6184), for a weighted average of 46% of adolescents doing some type of PA with their parents. Girls and boys reported walking with their parents once a week or more at similar rates (both 54%). Among both younger and older adolescents, boys and girls reported similar frequencies of walking with parents once a week or more (60% in younger adolescents and 44% in older adolescents). The grading of this indicator was limited in that only one of the benchmarks was used, and at the same time, was graded based on data from children rather than parents. Gaps in data were observed in the data gathered directly from parents regarding the facilitation of PA in their children, PA of the parents (either self-reported or device-measured), and for the children.

### School (B)

The average percentage for the *School* indicator was calculated based on various benchmarks. Physical education (PE) is mandatory in all schools in Slovakia, with two mandatory PE classes per week and one optional class if the school decides to incorporate it. Therefore, 100% of schools offered the mandated amount of PE. The situation with PE specialists was less favourable, as only 80.2% of teachers teaching PE were qualified PE specialists in 2014 [39]. The infrastructure of schools has been of concern to agencies such as the State School Inspection and Ministry of Education, Science, Research, and Sport of the Slovak Republic for some time now, as only 78% of schools have facilities for PA [40]. In addition, the proportion of 10-to 17-year-olds who reported having spaces in school for PA and sports outside of PE class was 86% (HBSC unpublished data). Regarding opportunities for PA in addition to PE classes, 63% of schools offered non-curricular PAs, 34% offered courses or training, and 46% offered non-regular (one-time) PAs [33]. Therefore, the situation within this indicator can be improved. The RWG members are currently involved in two initiatives, including the legislative process of increasing the number of mandatory PE classes from two to three and incorporating the Active School concept [41] into the reformed school curricula in Slovakia. An initiative to improve school infrastructure by investing in the reconstruction of old facilities or the construction of new ones is also ongoing.

### Community and environment (B–)

Two benchmarks related to the availability of facilities, programmes, parks, and playgrounds in the community and the safety of the neighbourhoods were used to grade *the Community and Environment* indicator. According to unpublished data from the HBSC, 60% (*whole sample size* = 1052) of 10- to 17-year-old adolescents reported that quality and appropriate activities or conditions were available in their neighbourhood, 77% (*whole sample size = 1056*) reported the availability of playgrounds or parks where they can play, 73% (*whole sample size = 1060*) reported they felt safe walking or playing during the day in their neighbourhood, and 58% (*whole sample size* = 5757) reported that it was safe for small children to play on the street during the day. The weighted average of the aforementioned data was 67% (68% for boys and 65% for girls). The main limitation in grading this indicator was the use of only some benchmarks and use of questionnaire data only from the adolescents. Since gaps in data from parents, communities/municipalities, and children were identified, we recommend focusing on these groups in further surveillance efforts. Surveillance methods should also include specific criteria that could be used by communities/municipalities for self-evaluation with respect to the infrastructure specifically geared towards promoting PA.

### Government (B–)

Nine policy instruments were identified and analysed against the six criteria using the scoring rubric published by Ward et al. (2021) [22], [42–50]. Although observable efforts are apparent, including action plans, legislation, concepts, and strategies to promote PA in children and adolescent, no evidence exists regarding the impact of these policies on PA levels or increases. Therefore, the lack of policy evaluation and accountability was identified as a major gap in the surveillance data for this indicator.

### Sleep (C–)

*Sleep*, as an additional indicator, was classified according to unpublished self-reported data from the HBSC (*n* = 8697; 9–17 years old) and according to the age-specific sleep time duration recommendations of the National Sleep Foundation. In general, 48% of children and adolescents met the recommended amount of sleep per night. Within both age categories, boys and girls had similar compliance rates with the aforementioned recommendations (42% vs. 40% in children < 13 years old and 55% vs. 53% in children > 14 years old). Based on these data, half of the adolescents did not meet the sleep duration recommendation. A surveillance gap was identified in the data for the other two benchmarks related to sleep quality as well as a gap in the data for younger children, thereby limiting the grading of this indicator. Simultaneously, a surveillance gap in the device-measured sleep duration data was identified.

### Practical applications and future research

Following the structure used by Tremblay et al. [17] we can outline various practical applications of the RC in Slovakia in different areas (Table 2).

**Table 2:** Practical applications of the Slovak Report Card

*Capacity building*	Firstly, fundraising activities aimed at ensuring the financing of ongoing activities within the AHKGA-GM project are being considered. Secondly, AHKGA-GM RC methodology would allow for comprehensive, periodical evaluation of PA surveillance system development in Slovakia. Thirdly, participation in development of AHKGA-GM RC may allow for personally rewarding experiences for participating experts by developing their own expertise and cooperation opportunities within the networks created within the project’s activities. And finally, usage of RC in the pre-service preparation of students within various study programmes (e.g. bachelor and master study programmes Sport for Health and doctoral study programme Sports Educology at the Faculty of Sports, University of Prešov) and/or their participation in RC development within their bachelor, master or dissertation thesis.
*Advancing knowledge*	Publication of the RC and planned preparation of infographics may potentially lead to advancing the knowledge about the surveillance data on PA behaviours, outcomes, and influencing factors among Slovakian children and adolescents in various stakeholders.
*Information decision*	RC may influence policy making by raising awareness on children’s and adolescent’s physical inactivity and may be used in the process of policy making considering all PA behaviours, outcomes, and influencing factors reported in RC.
*Health*	RC may indirectly influence health behaviours and consequently health outcomes of Slovak children and adolescents by contributing to development of quality PA surveillance system in Slovakia. Paraphrasing the quote of Friel, Vlahov and Buckley [51] „*No Data, No Problem, No Action.*“, quality surveillance data may lead to better problem description and thus to appropriate actions proposals.
*Economic and social benefits*	RC may have indirect economic and social benefits by indirectly influencing health behaviours and consequently health outcomes of Slovak children and adolescents. The appropriate prevention and control measures and health promotion strategies based on the quality surveillance data [52] may lead to economic investments in PA which, as evidence shows, can generate a positive (non)monetary return on investment [53].
*Social engagement*	RC in a form of this study and planned infographics may reach wider audience of various stakeholders and thus disseminate the outcomes of the AHKGA-GM project.

Identified surveillance gaps are important for planning of future research activities in Slovakia in order to enhance surveillance system. Since most of the available data come from adolescents via HBSC study (age group 10–15(17)), the focus should be put as well on younger children and parents. The study like WHO’s European Childhood Obesity Surveillance Initiative (COSI; age group 6–9 years) can potentially serve as a valuable complementary sources of quality surveillance PA data.

At the same time, since all available data was questionnaire-based, the future research should focus on the usage of device-based measurements of the movement behaviours in children, adolescents and their parents. The first such a research using accelometry was conducted in reaction to our findings in the sample of 100 adolescents of one high-school in 2022 in Prešov, Slovakia (no data published yet).

Further research activities should be focused on Active Play indicator widening the scope of such a research to outdoor physical activities. In reaction to our findings, there were two grant applications submitted within two Slovak national grant agencies (one scientific and one educational) in 2022, and, another grant application is currently under preparation within the EU’s Interreg programme. Furthermore, research cooperation within the European Network of Outdoor Sports (ENOS) was initiated, and RC Country leader became member of the steering committee of the 10-Year Anniversary of the Position Statement on Active Outdoor Play [54].

Finally, future research should focus on policy evaluation which can help improve public policies for the promotion of PA.

### Strengths and limitations

The study’s strengths include a usage of harmonised methodology used by the 57 countries and regions that participated in the AHKGA-GM 4.0 project. Another strength is usage of comprehensive multilevel data search strategy which allowed for extensive identification of relevant data resources. Two study limitations need to be mentioned. Firstly, varied surveillance methods used among countries included in the AHKGA-GM project make comparisons among countries challenging and often invalid. Therefore, these results should be considered with caution. Secondly, the variability of data resources did not allow for usage of any other quality assessment tool and therefore non-standardised tool was used. And, thirdly, numerous surveillance gaps were identified, which limit the overall informative value of the current grade.

## Conclusions

This comprehensive analysis is the first of its kind in Slovakia, and is intended to serve as an advocacy tool for PA promotion in Slovakian children and adolescents through the analysis of surveillance data on various PA-related indicators, comparisons with international data, and identification of surveillance gaps. The overall grading of the available surveillance data suggests the need for improvement in all 10 surveilled core indicators, as well as the additional *Sleep* indicator. Despite the fact that numerous identified surveillance gaps limit the overall informative value of the current grade, they provide the important information needed to enhance surveillance of PA-related indicators in Slovakia. For instance, the focus should be put as well on younger children and parents, on obtaining the device-measured data on various movement behaviours, on the topic of outdoor physical activities and policy evaluation.

### Electronic supplementary material

Below is the link to the electronic supplementary material.

**Additional file 1: Additional table 1.** List of data resources

**Additional file 2: Additional table 2.** Global Matrix 4.0 indicators, definitions, and benchmarks used to guide the grade assignment process

## Data Availability

The datasets generated and analysed during the current study are not publicly available, although can be requested from the corresponding author.

## Abbreviations

AHKGA-GM: Active Healthy Kids Global Alliance Global Matrix
COSI: WHO’s European Childhood Obesity Surveillance Initiative
CRT: Central Registry of Thesis
ENOS: European Network of Outdoor Sports
GAPPA: Global Action Plan on Physical Activity 2018–2030
HBSC: Health Behaviour in School-Aged Children Study
HDI: Human Development Index
PA: Physical activity
PC: Personal computer
PE: Physical education
RC: Report Card
RWG: Research work group
WHO: World Health Organization
