# Supplementary material for: First report card on physical activity for children and adolescents in Slovakia: a comprehensive analysis, international comparison, and identification of surveillance gaps
Source: Arch Public Health. 2024 Jan 30;82:16. doi: 10.1186/s13690-024-01241-4 (PMC10826129; doi:10.1186/s13690-024-01241-4)
Supplement: Supplementary file 1 — Additional file 1: Additional table 1. List of data resources [file 13690_2024_1241_MOESM1_ESM.docx]

| **Additional table 1**. List of data resources | | | | | | | | | | | | | | |
| --- | --- | --- | --- | --- | --- | --- | --- | --- | --- | --- | --- | --- | --- | --- |
| Guarantor/data custodian  (Institution, name of the study) | Status | Data collection | Sample  size | Data availability | | | | | | | | | | |
|  |  |  |  | 1 | 2 | 3 | 4 | 5 | 6 | 7 | 8 | 9 | 10 | 11 |
| Bakalár, P. (UNIPO, HBSC) | Published^24^ | 2018 | 8710 | ☑ |  |  |  |  |  |  |  |  |  |  |
| Bakalár, P. (UNIPO, HBSC) | Unpublished | 2018 | 8574 |  | ☑ |  |  |  |  |  |  |  |  |  |
| Bakalár, P. (UNIPO, HBSC) | Unpublished | 2018 | 8392 |  | ☑ |  |  |  |  |  |  |  |  |  |
| Bakalár, P. (UNIPO, HBSC) | Unpublished | 2018 | 1062 |  |  |  | ☑ |  |  |  |  |  |  |  |
| Bakalár, P. (UNIPO, HBSC) | Unpublished | 2018 | 1053 |  |  |  |  | ☑ |  |  |  |  |  |  |
| Bakalár, P. (UNIPO, HBSC) | Unpublished | 2018 | 1052 |  |  |  |  | ☑ |  |  |  |  |  |  |
| Bakalár, P. (UNIPO, HBSC) | Unpublished | 2018 | 6179 |  |  |  |  |  |  | ☑ |  |  |  |  |
| Bakalár, P. (UNIPO, HBSC) | Unpublished | 2018 | 6184 |  |  |  |  |  |  | ☑ |  |  |  |  |
| Bakalár, P. (UNIPO, HBSC) | Unpublished | 2018 | - |  |  |  |  |  |  |  | ☑ |  |  |  |
| Bakalár, P. (UNIPO, HBSC) | Unpublished | 2018 | 1052 |  |  |  |  |  |  |  |  | ☑ |  |  |
| Bakalár, P. (UNIPO, HBSC) | Unpublished | 2018 | 1056 |  |  |  |  |  |  |  |  | ☑ |  |  |
| Bakalár, P. (UNIPO, HBSC) | Unpublished | 2018 | 1060 |  |  |  |  |  |  |  |  | ☑ |  |  |
| Bakalár, P. (UNIPO, HBSC) | Unpublished | 2018 | 5757 |  |  |  |  |  |  |  |  | ☑ |  |  |
| Bakalár, P. (UNIPO, HBSC) | Unpublished | 2018 | 8697 |  |  |  |  |  |  |  |  |  |  | ☑ |
| Čillík, I. (UMB) | Published^34^ | 2014/2015 | 2006 |  |  |  |  |  | ☑ |  |  |  |  |  |
| Horváth, R. (UNIPO) | Published^36^ | 2016/2017 | 190 |  |  |  |  |  | ☑ |  |  |  |  |  |
| Act no 310/2019 Coll. | Published^50^ | 2019 | - |  |  |  |  |  |  |  |  |  | ☑ |  |
| Act no 440/2015 Coll. | Published^45^ | 2015 | - |  |  |  |  |  |  |  |  |  | ☑ |  |
| Madarasová Gecková, A. (UPJŠ, HBSC) | Published^33^ | 2018 | - |  |  |  |  |  |  |  | ☑ |  |  |  |
| MESRS | Published^39^ | 2014 | - |  |  |  |  |  |  |  | ☑ |  |  |  |
| MESRS | Published^43^ | 2014 | - |  |  |  |  |  |  |  |  |  | ☑ |  |
| MESRS | Published^46^ | 2015 | - |  |  |  |  |  |  |  |  |  | ☑ |  |
| MESRS | Published^42^ | 2012 | - |  |  |  |  |  |  |  |  |  | ☑ |  |
| MT | Published^48^ | 2015 | - |  |  |  |  |  |  |  |  |  | ☑ |  |
| Public Health Authority | Published^49^ | 2017 | - |  |  |  |  |  |  |  |  |  | ☑ |  |
| Public Health Authority | Published^47^ | 2015 | - |  |  |  |  |  |  |  |  |  | ☑ |  |
| Public Health Authority | Published^44^ | 2014 | - |  |  |  |  |  |  |  |  |  | ☑ |  |
| Novotná, B. (KU) | Published^27^ | 2017 | 625 |  |  | ☑ |  |  |  |  |  |  |  |  |
| Rozim, R. (UMB) | Published^35^ | 2017 | 306 |  |  |  |  |  | ☑ |  |  |  |  |  |
| SCSTI | Unpublished | 2018 | - |  |  |  |  |  |  |  | ☑ |  |  |  |
| Kováč, I. (SOSC) | Published^37^ | 2019/2020 | 1707 |  |  |  |  |  | ☑ |  |  |  |  |  |
| Note: Some of the listed resources were used as data resources of multiple benchmarks; UNIPO – University of Prešov; HBSC – Health Behaviour in School-aged Children; PJŠU – Pavol Jozef Šafárik University; UMB – University of Matej Bel; MESRS – The Ministry of Education, Science, Research and Sport of the Slovak Republic; MT – The Ministry of Transport of the Slovak Republic; SCSTI - Slovak Centre of Scientific and Technical Information; SOSC – Slovak Olympic and Sport Committee ; 1 – *Overall Physical Activity*; 2 – *Organized Sport and Physical Activity*; 3 – *Active Play*; 4 – *Active Transportation*; 5 – *Sedentary Behaviour*; 6 – *Physical Fitness*; 7 – *Family and Peers*; 8 – *School*; 9 – *Community and Environment*; 10 – *Government*; 11 – *Sleep* | | | | | | | | | | | | | | |
