# Supplementary material for: First report card on physical activity for children and adolescents in Slovakia: a comprehensive analysis, international comparison, and identification of surveillance gaps
Source: Arch Public Health. 2024 Jan 30;82:16. doi: 10.1186/s13690-024-01241-4 (PMC10826129; doi:10.1186/s13690-024-01241-4)
Supplement: Supplementary file 2 — Additional file 2: Additional table 2. Global Matrix 4.0 indicators, definitions, and benchmarks used to guide the grade assignment process [file 13690_2024_1241_MOESM2_ESM.docx]

| **Additional table 2.** Global Matrix 4.0 indicators, definitions, and benchmarks used to guide the grade assignment process | | | |
| --- | --- | --- | --- |
| **Indicator** | **Definition** | **Benchmark** | **Data Availability** |
| *Overall Physical Activity* | Any bodily movement produced by skeletal muscles that requires energy expenditure. | Percentage of children and adolescents who met the Global Recommendations on Physical Activity for Health, such as the accumulation of an average of at least 60 minutes of moderate-to vigorous intensity PA per day. | No |
|  |  | Percentage of children and adolescents who met the guidelines for at least 4 days/week (when the average cannot be estimated). | Yes |
| *Organised Sports and Physical Activity* | A subset of PA that is structured, goal oriented, competitive, and contest based. | Percentage of children and adolescents who participated in organised sports and/or PA programs. | Yes |
| *Active Play* | Active play may involve symbolic activity or games with or without clearly defined rules; the activity may be unstructured, unorganised, social, or solitary, although the distinguishing features are a playful context, combined with activity that is significantly above resting metabolic rate. Active play tends to occur sporadically, with frequent rest periods, making it difficult to record. | Percentage of children and adolescents who engaged in unstructured/unorganised active play at any intensity level for more than 2 hours/day. | No |
|  |  | Percentage of children and adolescents who reported being outdoors for more than 2 hours/day. | Yes |
| *Active*  *Transportation* | Active transportation refers to any form of human-powered transportation such as walking, cycling, using a wheelchair, in-line skating, or skateboarding. | Percentage of children and adolescents who used active transportation (e.g. to school, park, mall, or friend’s house). | Yes |
| *Sedentary Behaviour* | Any waking behaviour characterised by energy expenditure ≤ 1.5 metabolic equivalents while in a sitting, reclining, or lying posture. | Percentage of children and adolescents who met the Canadian sedentary behaviour guidelines (5–17-year-olds: no more than 2 hours of recreational screen time per day). Note: The Guidelines currently recommend a limit for screen-related pursuits, although not for non-screen-related pursuits. | Yes |
| *Physical Fitness* | Characteristics that permit good performance of a given physical task in a specified physical, social, and psychological environment. | Average percentile achieved on certain physical fitness indicators based on the normative values published by Tomkinson et al. (2018)*. | Yes |
| *Family and Peers* | Any member within the family who can control or influence the opportunities for and participation in PA by children and adolescents in this environment. | Percentage of family members (e.g. parents, guardians) who facilitated PA and sports opportunities for their children  (e.g. volunteering, coaching, driving, paying for membership fees and equipment). | No |
|  |  | Percentage of parents who met the Global Recommendations on Physical Activity for Health, which recommend that adults accumulate at least 150 minutes of moderate-intensity aerobic PA throughout the week or to perform at least 75 minutes of vigorous-intensity aerobic PA throughout the week or an equivalent combination of moderate- and vigorous-intensity PA. | No |
|  |  | Percentage of family members (e.g. parents, guardians) who were physically active with their kids. | Yes |
|  |  | Percentage of children and adolescents with friends and peers who encouraged and supported them to be physically active. | No |
|  |  | Percentage of children and adolescents who encouraged and supported their friends and peers to be physically active. | No |
| *School* | Any policies, organisational factors (e.g. infrastructure, accountability for policy implementation), or student factors (e.g. PA options based on age, sex, or ethnicity) in the school environment that can influence the opportunities for and participation in physical activity by children and adolescents in this environment. | Percentage of schools with active school policies (e.g. daily PE, daily PA, recess, ‘everyone plays’ approach, bike racks at school, traffic calming on school property, outdoor time). | No |
|  |  | Percentage of schools where the majority (≥ 80%) of students were taught by a PE specialist. | Yes |
|  |  | Percentage of schools where the majority (≥ 80%) of students were offered the mandated amount of PE (for the given state/ territory/region/country). | Yes |
|  |  | Percentage of schools that offered PA opportunities (excluding PE) to the majority (> 80%) of their students. | Yes |
|  |  | Percentage of parents who reported their children and adolescents had access to PA opportunities at school in addition to PE classes. | No |
|  |  | Percentage of schools with students who had regular access to facilities and equipment that support PA (e.g. gymnasium, outdoor playgrounds, sporting fields, multipurpose space for PA, equipment in good condition). | Yes |
| *Community and Environment* | Any policies or organisational factors (e.g. infrastructure, accountability for policy implementation) in the municipal environment that can influence opportunities for and participation in physical activity by children and adolescents in this environment. | Percentage of children or parents who perceived their community/ municipality as doing a good job at promoting physical activity (e.g. variety, location, cost, quality). | No |
|  |  | Percentage of communities/municipalities that reported the presence of existing policies that promote PA. | No |
|  |  | Percentage of communities/municipalities that reported the presence of infrastructure (e.g. sidewalks, trails, paths, bike lanes) specifically geared toward the promotion of PA. | No |
|  |  | Percentage of children or parents who reported having facilities, programs, parks, and playgrounds in their community. | Yes |
|  |  | Percentage of children or parents who reported living in a safe neighbourhood in which they can be physically active. | Yes |
|  |  | Percentage of children or parents who reported having safe, well-maintained facilities, parks, and playgrounds in their community. | No |
| *Government* | Any governmental body with the authority to influence opportunities for or participation in physical activity by children and adolescents through policy, legislation, or regulations. | Evidence of leadership and commitment in providing PA opportunities for all children and adolescents. | Yes |
|  |  | Allocated funds and resources for the implementation of PA promotion strategies and initiatives for all children and adolescents. | Yes |
|  |  | Demonstrated progress through the key stages of public policy making (i.e. policy agenda, formation, implementation, and evaluation and decisions about the future). | Yes |
| *Sleep* |  | Percentage of children and adolescents who met the recommended number of sleep hours per night according to the National Sleep Foundation guidelines (9–11 hours for children, 8–10 hours for adolescents) [23]. | Yes |
|  |  | Percentage of children and adolescents with poor sleep quality. | No |
|  |  | Percentage of children and adolescents who reported sleep disturbances. | No |
| Note: The Sleep indicator is in addition to the 10 core indicators; PA – Physical Activity; PE – Physical Education; *Tomkinson GR, Carver KD, Atkinson F, Daniell ND, Lewis LK, Fitzgerald JS, et al. European normative values for physical fitness in children and adolescents aged 9–17 years: results from 2 779 165 Eurofit performances representing 30 countries. Br J Sports Med. 2018;52:1445–56. | | | |
